# Inverted ILM Flap for a Needle Injury to the Macula after Peribulbar Anaesthesia: A Case Report and Literature Review

**DOI:** 10.3390/life13061390

**Published:** 2023-06-14

**Authors:** Alessandra Scampoli, Lorenzo Governatori, Patrizio Bernardinelli, Stefano Maria Picardi, Carola Culiersi, Tomaso Caporossi

**Affiliations:** 1 Vitreoretinal Surgery Unit, Fatebenefratelli Isola Tiberina-Gemelli Isola Hospital, 00186 Rome, Italy; lorenzo.gov@gmail.com (L.G.); tomaso.caporossi@gmail.com (T.C.); 2 Catholic University of the Sacred Heart, 00168 Rome, Italy; patrizio.bernardinelli@gmail.com (P.B.); c.carola@live.it (C.C.); 3 Ophthalmology Unit, Fondazione Policlinico Universitario A. Gemelli IRCCS, 00168 Rome, Italy; 4 Ophthalmology Unit, ASST Melegnano e Della Martesana, 20070 Vizzolo Predabissi, Italy; ste.picardi@gmail.com

**Keywords:** rhegmatogenous retinal detachment, vitrectomy, vitreoretinal surgery, peribulbar anesthesia, needle injury, macular break, inverted flap, ocular perforation, vitreous hemorrhage

## Abstract

Globe perforation following peribulbar anesthetic injection is a rare but dreaded complication that often results in poor visual outcomes. This case report is on a female patient who sustained vitreous hemorrhage, retinal detachment, and macular breaks due to a peribulbar block administered in the setting of cataract extraction. The retina was repaired with pars plana vitrectomy, endolaser of the peripheral retinal break only, and an internal limiting membrane inverted flap for the macular breaks to avoid the endolaser on the macular area, achieving stable visual outcomes. The authors discussed various modes of local anesthesia for vitreoretinal surgery, risks for globe perforations, and how to approach retinal detachment secondary to needle perforations, which are complex cases at high risk for proliferative vitreoretinopathy. Early recognition and intervention in eyes with an inadvertent perforation can lead to a good outcome. Eyes with a longer axial length, superior, and multiple perforations are at higher risk of developing complications such as retinal detachment and vitreous hemorrhage. Complications such as retinal detachment, macular injury, and vascular occlusion are risk factors for poor prognosis.

## 1. Introduction

Peribulbar anesthesia (PBA) is an extraconal regional block that was introduced by Davis and Mandel in the 1970s [1] and is used when akinesia is required in addition to analgesia. Compared to the intraconal retrobulbar technique, PBA is safer and easier, so it has gained popularity in recent years, as has the sub-tenon block. With a 25 mm needle, PBA can reach sufficient anesthesia, akinesia, and analgesia with a single or double injection above and below the orbit, acting on ciliary nerves and cranial nerves III and VI [1]. Although rare, different complications are described in the literature [2], ranging from minor lid edema and chemosis to grave events such as optic nerve damage, extraocular muscle damage, retrobulbar hemorrhage, central retinal artery occlusion, and globe penetration. Inadvertent globe penetration during peribulbar block is considered a very rare event with a prevalence between 1 in 1000 and 1 in 10,000 cases [2,3].

Described risk factors for globe penetration include posterior staphyloma [4], long axial length [5], inexperienced personnel [6], uncooperative patients [7], the use of sharp long needles, and the use of multiple injections [8]. Regarding the described prognosis factors, outcomes are related to (1) early recognition, (2) absence of retinal detachment (RD), and (3) site of penetration [8,9]. In this work, we reported a case of retinal detachment with posterior pole laceration after PBA for cataract surgery in a hyperopic patient, who was referred to the Vitreoretinal Surgical Unit of Fatebenefratelli-Gemelli Isola. We also reported details of the clinical features, management, and subsequent outcomes.

## 2. Case Report

This study examined a case in the Ophthalmology Unit of Fatebenefratelli-Gemelli Isola, Rome, in November 2022. Approval from our institutional review board was obtained for the retrospective review of the patient’s clinical records. This study was conducted in accordance with the tenets of the Declaration of Helsinki.

A 55-year-old woman was scheduled for a clear lens extraction and intraocular lens (IOL) implantation with an emmetropic target. She had a preoperative best corrected visual acuity (BCVA) of 20/20, a hyperopic correction of +3.00 dioptres, and a shallow anterior chamber with a familiar history of glaucoma. She had already undergone cataract surgery in the other eye a few months before. The right eye had an axial length (AL) of 21.62 mm, and she planned for a +27.0-diopter IOL implantation. The other eye (the left eye) was pseudophakic, and its BCVA was 20/20.

Before the surgery, the surgeon performed a peribulbar anesthesia (PBA) with a 25 G × 25 mm sharp-edge needle in the infero-temporal quadrant. During the first injection, the surgeon reported an abnormal resistance to the injection of the anesthetic that led to halting the maneuver and the withdrawal of the needle. A new injection site to perform the anesthesia was found. The cataract surgery was performed uneventfully with standard IOL implantations. The surgeon reported an abnormal loss of the red fundus reflex during the surgery, which was not significant enough to halt the surgery.

On the first post-operative day, the patient’s visual acuity was light perception, there was no pain, and the intraocular pressure (IOP) was 16 mmHg. The anterior segment had a pseudophakic status of the lens, and the dilatated fundus was not explorable due to a massive vitreous hemmorhage (HV). B-scan ultrasonography showed a massive vitreous hemorrhage with a bullous retinal detachment (RD) localized in the infero-temporal quadrant of the retina and a subretinal hemorrhage (SRH).

The patient was immediately referred to our surgical department, and on the same day, a surgeon (T.C.) performed a standard 23-gauge pars plana vitrectomy (PPV) (Alcon, Fort Worth, TX, USA) with the Constellation^®^ and NGENUITY^®^ 3D Visualization System (Alcon, Fort Worth, TX, USA) under a peribulbar block. After careful hemovitreous removal, an inferior RD involving two quadrants was observed with a retinal laceration and a subretinal blood clot. There were also two large retinal breaks in the macular area temporally and inferiorly to the fovea. The fovea and the optic nerve were apparently healthy. We could hypothesize that during the first injection, the needle penetrated the eye bulb in the inferotemporal quadrant and impacted the macula at the end of the stroke.

After a complete vitreous base trimming to remove all the peripheral tractions and the remnants of hemorrhage, we injected a combined vital dye (Membraneblue-Dual^®^, DORC International, Zuidland, The Netherlands), followed by perfluoro-N-octane (PFC) (EFTIAR Octane^®^, DORC International, Zuidland, The Netherlands) in the macular area. Internal limiting membrane (ILM) peeling was performed in the macula using end gripping vitreous forceps (Greishaber, Alcon Laboratories, Fort Worth, Texas) to create two big ILM flaps to cover the posterior breaks and avoid laser retinopexy in the macular area. The PFC was injected until the margin of the peripheral retinal broke, and the blood clot was removed from the subretinal space through the retinal break. A fluid–air exchange was performed, and laser retinopexy was applied to only the site of the infero-temporal retinal break. Additionally, 20% sulfur hexafluoride (GOT SF6 Multi^®^, Alchimia Srl, Padua, Italy) was chosen as an endotamponade. The patient was kept in a face-down position for 5 days postoperatively.

**Figure 1:**
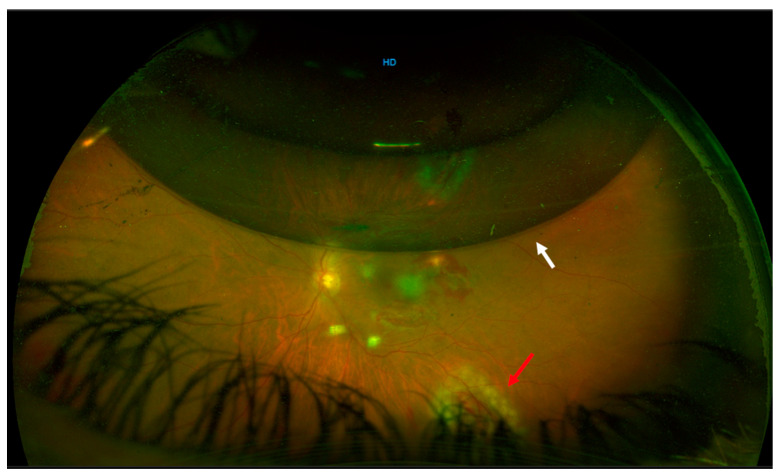
One week after surgery, the gas bubble was at 50% size (white arrow) with an attached retina and endolaser spots at the site of the retinal break (red arrow).

**Figure 2:**
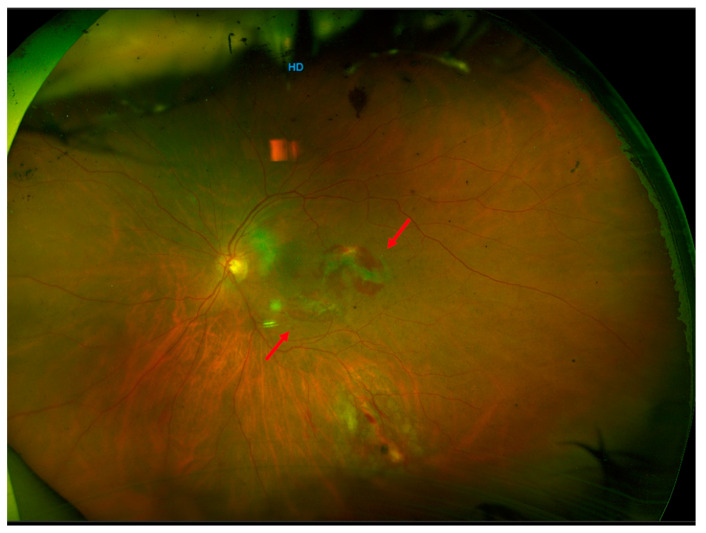
Three weeks after surgery, the gas was completely resorbed, and the retina was completely reattached. The macular retinal breaks remained sealed without laser retinopexy (red arrow).

**Figure 3:**
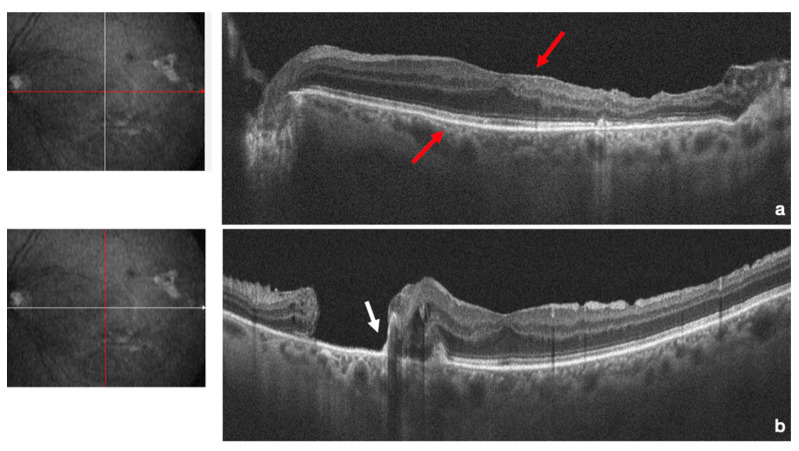
Structural optical coherence tomography shows that the structure of the fovea had returned to normal (red arrow) (**a**) with external retinal layers, and the posterior breaks were sealed with the ILM flap which lay on the bottom of the break (white arrows) (**b**).

After the earlier surgical intervention, successful reattachment of the retina was achieved. One week after surgery, the gas bubble was at 50% size with an attached retina and endolaser spots at the site of the retinal break (Figure 1). Three weeks after surgery, the gas was completely resorbed, the retina had completely reattached, and the patient’s BCVA improved to 20/32 (Figure 2). Further fundus examinations and ultra-wide field retinography (Optomap, Optos Inc. Marlborough, MA, USA) showed that the retina remained attached. Three months after surgery, the patient’s BCVA recovered to 20/20. Structural optical coherence tomography (AngioVue Optovue, Fremont, CA, USA) revealed that the structure of the fovea had returned to normal, and the posterior breaks were sealed with the ILM flap alone (Figure 3) (Supplementary Video S1).

## 3. Discussion

Cataract surgery is the most frequent surgical procedure in ophthalmology and surgeons have the opportunity to choose from different techniques of anesthesia, which make them more comfortable with the surgery. Needle block has a rare but real rate of feared complications, for which careful training is required to avoid needle misplacement. In the past, the most performed anesthesia was the retrobulbar block (RBA), an intraconal procedure formally described in 1936 by Atkinson, which consisted of injecting a small amount (3–5 mL) of an anesthetic drug inside the muscular cone. Both the globe and the ocular structures located in the muscular cone lie in a small and compressed space, for which passing the needle near them increases the risk of damage and complications. RBA has been progressively replaced by peribulbar block (PBA), apparently less dangerous, sub-Tenon’s block (STB), and topical anesthesia (T).

Nowadays, PBA is the most popular regional block performed during ophthalmological procedures, and if topical cataract surgery is performed, it is still used often for complicated cases. PBA is an extraconal regional block that was introduced by Davis and Mandel in the 1970s [1] to reduce the risk of injury of the intraconal structures. A greater volume of anesthetic drug is needed compared to RBA, up to 12 mL, to be able to spread more widely and reach both the corpus adiposum of the orbit and the eyelids, providing a block of the orbicular muscle. Normally, the PBA procedure requires one injection, inferiorly and temporally, that can be followed by a second one superiorly and nasally as a supplement when the first one has failed. An alternative site of a second puncture for PBA is the medial canthus, nasally to the lachrymal caruncle. The needle has to be introduced to a maximum depth of 15 mm. The space between the globe and the orbital wall is comparable to the space with the inferior and temporal approach, different from the superior and nasal site of puncture, where the distance between the globe and the orbital roof is reduced, increasing the risk of eye perforation.

Although it is commonly believed that RBA is more effective than PBA, both procedures have similar efficacies. In fact, there is no intramuscular septum that physically separates intraconal and extraconal spaces, allowing the diffusion of the local anesthetic in a single, unique space. If efficacy is similar, what differentiates the technique is the safety since RBA has a higher risk of complications (optic nerve injury, brainstem anesthesia, retrobulbar hemorrhage) [10].

Scleral perforation associated with retrobulbar anesthesia is more common than scleral perforation associated with peribulbar anesthesia [11] and inadvertent globe penetration during peribulbar block is considered a very rare event with a prevalence of 0.006–0.13%. When this complication is recognized, the patient may feel a sharp pain accompanied by a sudden loss of vision. Early signs of this serious complication include increased IOP and an abnormal red reflex [2,3].

Described risk factors for globe penetration include posterior staphyloma [4], long axial length [5], inexperienced personnel [6], uncooperative patients [7], the use of sharp long needles, and the use of multiple injections [8]. The risk of perforation is 10 to 30 times higher in highly myopic eyes with axial lengths ≥ 26.0 mm that are otherwise normal [11]. To reduce the risk of globe penetration in myopic eyes, it is advisable to use a B-ultrasound examination to exclude staphyloma at the injection site preoperatively. Recently, Foad et al. explored the accuracy and safety of real-time ultrasound-guided retrobulbar regional anesthesia for cataract surgery and reported that ultrasound-guided retrobulbar injection may decrease the risk for sight-threatening or life-threatening complications [12]. However, our patient had no high-risk factors, and in this case, one possible explanation was that the surgeon applied an incorrect angle of needle insertion.

Some features of the administration technique of the anesthetic drugs, such as the use of sharp long needles and the use of multiple injections, have been described as risk factors for ocular penetration [9]. In our case, peribulbar anesthesia with a 25-gauge 25 mm sharp needle was used. Previous studies reported globe penetration using blunt needles without statistical differences. Hay et al. reported the same percentage of globe perforation with a blunt o sharp needle with no statistically significant difference [13]. However, it is believed that shorter needles may reduce needle-related perforation. In fact, a 3.8 cm needle was used in most published studies. In the last few years, shorter 2.5 cm needles have been used [14], while some authors claim excellent results with a 1.5 cm needle [15,16]. In our case, the needle used to perform the anesthesia increased the risk of perforation. Concerning the number of injections, Ball reported that a second injection may increase the risk of penetration especially if it is placed immediately after a first injection on the opposite side due to globe displacement [17]. However, in our case, the perforations happened during the first injection.

Liang investigated the toxicity of various concentrations of intravitreal lidocaine, bupivacaine, and a combination of both in animal models. Reversible changes were observed in electroretinography. No permanent histologic abnormalities were observed [18]. After the intravitreal injection of lidocaine, no functional or histological changes were noted in the retinas of rabbits [19] Olmez reported the effect of intravitreal ropivacaine on retinal thickness and integrity in animal models. Histopathological analysis revealed structural abnormalities in the retinas of eyes that received 0.1 mL of 0.75% or 1% ropivacaine [20] In this situation, a prompt PPV should be considered to minimize the retinal toxicity caused by anesthetics.

Ocular penetration can lead to many serious complications such as retinal breaks, vitreous hemorrhage (VH), subretinal hemorrhage (SRH), and RD [21] Identifying a globe perforation at the time of the injection is not easy. A previous paper reported that globe perforations were noticed in 18 of 20 eyes within one week after, and such as our case, globe perforation was identified on the first day after surgery due to VH in 15 eyes [2].

Alrajhi et al. [22] reported a case of globe penetration after a PBA for cataract surgery, and on the day after surgery, it showed VH and RD. Differently from our case, they reported no macular injury but found optic disk damage, probably due to the injection of the anesthetic inside the globe, with a final BCVA of light perception even after retinal reattachment. In our case, the surgeon was unable to inject the anesthetic inside the eye because the tip of the needle was blocked by the surface of the retina with which it had impacted.

Gopal et al. [23] reported five cases of RD after globe perforation for PBA. Differently from our case, which was hyperopic, all the cases reported were highly myopic eyes with a higher risk of globe perforation due to the scleral staphyloma [4,5,6,13] Gomez-Benlloch et al. reported a percentage of 0.024% (4 of 17,460 eyes) [9]. Two of the four cases reported were highly myopic, and two cases developed RD complicated by choroidal detachment. In our case, massive VH was observed, and a diagnosis of RD and SRH was made with B-scan ultrasonography.

SRH may contribute to photoreceptor damage and vision loss by exerting toxic effects on choriocapillaris circulation, leading to photoreceptor apoptosis. Moreover, during clot retraction, fibrin may contribute to retinal traction and proliferative vitreoretinal (PVR) formation [24] In the literature, a worse visual outcome is reported in patients with RD than those without RD [5,13] Hay et al. reported that a final BCVA > 20/400 was achieved in 14.3% of patients with RD [13]. Duker [5] reported that a final BCVA > 20/400 was achieved in 9.1% of patients with RD. Wearne et al. [25] reported that 78% of patients had poorer BCVA than counting fingers with RD. Babu et al. [8] reported that 40% of patients with BCVA > 20/200 had RD.

Gomez-Benlloch et al. reported one case of four with a final BCVA of 20/20. The worst result was obtained in a patient who developed a PVR and a recurrence of RD. However, they all had good visual recovery due to the early recognition and treatment [9].

Regarding the inverted flap technique, the introduction of the use of layered tissue for retinal wound closure dates to 2010, when Michalewska published the idea of using an inverted ILM flap over idiopathic MHs [25]. The technique has been adopted all over the world and adapted for the treatment of larger and chronic idiopathic MH, myopic MH, and, lastly, posterior retinal tears as we described in this case report [26,27].

## 4. Conclusions

The reported cause of poor final BCVA in eyes complicated with RD include epiretinal membrane formation, macular injury due to a needle, optic atrophy, SRH, CD, and vascular occlusion [26,27,28,29,30,31]. In our case, perforation signs such as VH, SRH, and RD were noted, leading to a high risk for PVR-complicated RD development. The reason why our case had a final BCVA of 20/20 despite the needle injury to the macula is linked to the sparing of the foveal area. Moreover, the peeling of the ILM and the inverted flap to cover the retinal breaks for sealing without laser retinopexy reduced the contraction of the posterior pole and the development of an epiretinal membrane, resulting in a healthy fovea. Early recognition and treatment are mandatory for a good outcome. B-scan ultrasonography is recommended in every suspicious case with dense VH.

## Supplementary Materials

The following supporting information can be downloaded at: https://www.mdpi.com/article/10.3390/life13061390/s1, Video S1: surgical technique.
